# A call to action for pharmacogenomics stewardship in South Africa

**DOI:** 10.3389/fphar.2026.1764125

**Published:** 2026-03-20

**Authors:** Nicole Keuler, Machel Leuschner, Tracey Hurrell, Renier Coetzee, Beatrice Jordaan, Werner Breedt, Natalie Schellack

**Affiliations:** 1 School of Pharmacy, University of the Western Cape, Cape Town, South Africa; 2 Department of Pharmacology, Faculty of Health Sciences, University of Pretoria, Pretoria, South Africa; 3 Bioengineering and Integrated Genomics Group, Future Production Chemicals Cluster, Council for Scientific and Industrial Research, Pretoria, South Africa; 4 School of Public Health, University of the Western Cape, Cape Town, South Africa

**Keywords:** education, implementation, pharmacogenomics, research, South Africa, stewardship

## Abstract

Pharmacogenomics (PGx) can personalize medication therapy in South Africa’s (SA) diverse population but faces context-specific barriers: inadequate SA-specific allele variant data, infrastructure limitations, education and training gaps, and economic constraints. This call to action proposes SA’s first PGx stewardship framework to guide rational PGx testing integration. Three critical pillars are as follows (1) targeted research to build an open-access PGx variant database informed by SA data; (2) phased implementation starting with high-risk medicines in tertiary hospitals; and (3) nationally accredited PGx curricula for health professionals. All pillars are grounded in culturally responsive ethical, legal and social frameworks addressing SA’s sociohistorical context. We advocate for interprofessional collaboration to position SA as a leader in equitable PGx implementation, aligning with National Health Insurance priorities to reduce adverse drug reaction (ADR) related hospitalizations by 30% within 5 years.

## 1 Introduction

Pharmacogenomics holds transformative potential for addressing South Africa’s dual burden of communicable and non-communicable diseases by optimizing medication safety and efficacy (Haskins, 2021; Soko et al., 2023). Clinical implementation of PGx can improve medication safety, reduce adverse drug reactions (ADRs), and optimize treatment efficacy, ultimately leading to better patient outcomes and more efficient healthcare resource utilization (David et al., 2021; Djuzic et al., 2023; Swen et al., 2023). As the global evidence base for effective implementation of PGx continues to grow, it presents a unique opportunity for South Africa to address disparities in healthcare access and enhance equitable treatment for diverse populations (Jordan et al., 2023). Pharmacogenomics may, once effectively established within a healthcare sector, offer a cost-effective strategy (Morris et al., 2022; Alimohamed et al., 2025) to reduce hospitalizations, improve adherence, and combat medication resistance (Haskins, 2021). However, context specific barriers like infrastructure and limited funding may inadvertently increase the costs in certain settings. While PGx may offer long-term cost savings, initial SA cost-benefit analyses are urgently needed. Early studies indicate that prioritization of PGx in South Africa may reduce adverse drug reactions related costs while advancing equitable precision medicine, especially in the context of a genetically diverse population (Hurrell et al., 2024; Smith et al., 2025).

## 2 Call to action and discussion

This call to action outlines the importance of rational, evidence-based implementation of PGx in clinical practice in South Africa to optimize medication therapy within both the public and private healthcare sectors. Here we will emphasize: 1) the potential of PGx to improve patient outcomes in South Africa; 2) the role of healthcare professionals in implementing and managing PGx testing and interpretation; 3) the alignment of PGx implementation with national health priorities; and 4) propose a framework for integrating PGx into existing healthcare systems.

### 2.1 Potential of pharmacogenomics in South Africa

Pharmacogenomics is a tool for healthcare professionals to practice personalized medicine (Kisor et al., 2016; Chart et al., 2021). Increasing evidence of the benefits of PGx support clinical implementation of actionable gene-drug pairs (FDA, 2023; Kabbani et al., 2023; Hurrell et al., 2024). Examples for rational medicine use to support patient safety across various therapeutic areas include cardiovascular diseases (Magavern et al., 2022; Duarte et al., 2024), psychiatric disorders (Bradley et al., 2018; Greden et al., 2019), pain management (Theken et al., 2020; Crews et al., 2021), HIV/tuberculosis (Martin et al., 2012), and oncology (Amstutz et al., 2018). The PREPARE study (Swen et al., 2023), conducted across seven European countries, demonstrated a 30% reduction in ADRs with pre-emptive pharmacogenetic testing. However, issues such as the open-label study design, inclusion of only 12 pharmacogenes, the interpretation and application of PGx recommendations, and the use of composite outcomes of ADRs may have impacted the quantification of ADR reduction. In addition, the limited ancestral diversity within the study population prevents generalizability of the findings to genetically diverse populations such as South Africa (e.g., African or Asian genetic ancestry). Although clinical benefit was shown, cost-effectiveness was not directly assessed in this study and health economic analyses are required to support implementation at scale. An individualized approach, especially in regions with more genetically diverse populations, could lead to cost-effective treatment (Dandara et al., 2014; Mitropoulos et al., 2015; Morris et al., 2022) and thereby promote health system efficiency by reducing adverse drug reactions and improving therapeutic efficacy. As an example, Sub-Saharan Africa has a 10% prevalence of HLA-B*58:01 which increases the population’s risk for severe cutaneous adverse reactions. Pre-emptive PGx testing could reduce the burden of ADRs in Africa (Karnes et al., 2019). Another prevalent gene in Africa is G6PD considering the high malaria burden and risk of mortality in G6PD deficient patients (Taleb et al., 2025; Kassahun, et al., 2023).

African populations are known for their genetic diversity, yet limited genetic data is currently available (Twesigomwe et al., 2025), with uneven geographic representation further limiting the accurate representation of the genetic diversity inherent to the continent (Radouani et al., 2020). Clinical implementation of PGx is not only impacted by a lack of knowledge of African genetics but factors such as phenocoversion need to be considered, especially considering Africa’s disease burden (Shah and Smith, 2015; Tata et al., 2020).

While PGx implementation has made notable progress in selected academic and private sector settings in developed countries (Jongeneel et al., 2022), the approach is not yet universally adopted. In Sub-Saharan Africa, academic pilot programs and targeted research initiatives have started exploring clinical translation (Mitropoulos et al., 2015; Twesigomwe et al., 2025), but widespread coverage remains limited. This is largely due to insufficient capacity, lack of infrastructure, limited clinical applicability, and gaps in genetic research and knowledge influencing disease and treatment response (Elewa and Awaisu, 2019; Tata et al., 2020; Twesigomwe et al., 2025). There are some unique initiatives and projects being conducted in Africa (Twesigomwe et al., 2025) such as the Human Heredity and Health in Africa (H3Africa) (Human Heredity and Health in Africa, 2023) and the African Pharmacogenetics Network (Dandara et al., 2019a) which are increasingly addressing research gaps in genomics representation and the implications thereof for health. The Department of Science, Technology and Innovation (DSTI) and the South African Medical Research Council (SAMRC) initiated a unique collaboration through the Strategic Health Innovation Partnerships (SHIP) initiative (Department of Science, Technology and Innovation, 2025). The South Africa 110 000 Human Genome Program aims to build a novel national genomic reference database enabling clinical research, data-driven public health strategies, biotechnology innovation and improved health equity.

Most PGx studies are conducted in developed countries, with less than 4% of data in PharmGKB representing African populations (Corpas et al., 2024). Therefore, the variants that inform evidence might not comprehensively represent the South African population, potentially perpetuating variable medication responses due to the unknown functional implications of different variants (Twesigomwe et al., 2025). Thus, context-specific research should be conducted to address the paucity in data and continuously update the local knowledge-base to ensure relevant practice. Variability in the allele frequencies of genes involved in the metabolism of medicines is driven by geographical ancestry, contributing to significant differences in treatment response, risk profiles, and disease prevalence (Zhou et al., 2017).

Access to context-specific, evidence-based data can provide integral knowledge on genetics variants in the population, potential novel variants in actionable genes, and support the discrete quantification of the impact on healthcare workers and patients (Smith et al., 2025). South African representative studies are limited (Kudzi et al., 2015; Muzoriana et al., 2017; Mufwambi et al., 2020; da Rocha et al., 2021). The ethical application of individualized medicine requires appropriate contextualization within local genetic variability. Considering our local disease burden and according to local researchers, the following proposed genes should be prioritized for further investigation to improve patient health outcomes in Southern Africa: ABCB1, CYP2B6, CYP2C9, CYP2C19, CYP2D6, CYP3A4, SLC22A1 and SLCO1B1 (Soko et al., 2023). More research is needed to further determine the actionability of these genes. Identifying population-specific pharmacogenomic alleles is a critical enabler for the effective and equitable implementation of PGx in South Africa. The South African population is characterized by exceptional genetic diversity (Corpas et al., 2025), and consequently, reliance on allele frequency data derived predominantly from European or Asian populations risks misclassification of metabolizer status. This may result in reduced predictive accuracy, and inequitable clinical benefit. Population-specific alleles directly influence the functional interpretation of drug-metabolizing enzymes, transporters, and drug targets. Several clinically relevant PGx variants, particularly within genes such as CYP2B6, CYP2D6, CYP2C9, CYP2C19, SLCO1B1, DPYD, and NAT2, display markedly different allele frequencies and structural variant patterns in African populations (Da Rocha et al., 2021). In some cases, novel or rare variants unique to African populations contribute substantially to interindividual variability in drug response but are absent from commonly used PGx panels (Tata et al., 2020). Knowledge of population-specific allele distributions enables the rational design of targeted PGx panels tailored to the South African population (Tata et al., 2020; Hurrell et al., 2024). Rather than deploying broad, costly panels designed for other populations, locally informed panels can prioritize variants with demonstrated clinical relevance and sufficient allele frequency to impact prescribing at a population level.

Pharmacogenomics holds various benefits for public health (Borbon et al., 2025) in South Africa by addressing critical challenges, such as hospital re-admissions in local healthcare systems. By including more diverse populations in research, we can contribute to the knowledge gap of relating genetic diversity to improve patient health outcomes in communicable and non-communicable diseases (Smith et al., 2025; Twesigomwe et al., 2025). Individualized medicine requires collaborative data sharing to increase the total value of global PGx outcomes. Offering PGx testing in both the public and private healthcare setting can further support population-relevant research insights thereby enhancing equity (Dandara et al., 2014) and access (Jongeneel et al., 2022) in healthcare.

The interplay of PGx, co-morbidities, and environmental or socio-economic factors, including the use of herbal and traditional medicines, can modify enzyme activity (Dandara et al., 2019b). This complexity underscores the need for more research in African settings to optimize the application of PGx in diverse and unique clinical contexts. Patients presenting with multiple comorbid conditions require a range of medicines, which could lead to various drug-drug interactions and phenoconversion. This poses a significant challenge to the interpretation of PGx test results. The phenomenon can also result from inflammatory processes, further complicating phenotype prediction (Shah and Smith, 2015). Despite its clinical importance, few studies globally, and even fewer in Africa, have explored the impact of phenoconversion on PGx test interpretation. Phenoconversion therefore highlights the limitations of isolated genotyping to guide individualized pharmacotherapy. This mismatch arises from complex interactions between genetic and non-genetic factors, underscoring the need for integrated approaches in clinical PGx (Shah and Smith, 2015; Dandara et al., 2019b; Klomp et al., 2020; Tata et al., 2020). Clinical PGx implementation must account for SA’s unique challenges: phenoconversion risks, and infrastructure limitations requiring tiered rollouts starting with NHLS-linked tertiary hubs.

### 2.2 The role of healthcare professionals in pharmacogenomics

Successful clinical PGx implementation is built on interprofessional collaboration, (Cicali et al., 2022; Gammal et al., 2022), stakeholder engagement, the healthcare setting, resources, insurance and organizational structures (Jongeneel et al., 2022; Kabbani et al., 2023). A core PGx team should consist at least of a physician, pharmacist, nurse, genetic counsellor, and laboratory specialists (Klein et al., 2017; Alhaddad et al., 2022). Healthcare providers can fulfil the following roles in PGx implementation: ordering the appropriate PGx test, interpreting results, optimizing medication therapy, PGx counselling, education of the interprofessional team and patients (Kabbani et al., 2023). However, healthcare professionals need to have adequate knowledge of relevant actionable gene-drug pairs in specific populations to ensure a positive impact on patient health outcomes. Healthcare providers play a critical role in participating in strategies to increase awareness among the public, healthcare professionals, and healthcare professional students. Education of healthcare professionals, healthcare professional students and the public is needed for each to have the relevant knowledge base to fulfil their role in supporting implementation (Gurwitz et al., 2005; Hicks et al., 2016; Haidar et al., 2022; Gammal et al., 2022). Competencies have been developed for various healthcare professionals which can assist higher education institutions to deliver graduates with knowledge and skills to serve the population (National Human Genome Research Institute, 2024). Table 1 below summarizes the various stakeholders to be involved in PGx implementation as informed by literature and authors’ recommendations.

**TABLE 1:** The role of PGx stakeholders and the rationale for their involvement in PGx implementation.

Stakeholder (Author)	Role	Rationale
Regulatory authority (Academy of Science of South Africa, 2018; Tata et al., 2020; Smith et al., 2025; Twesigomwe et al., 2025)	National policies to ensure equitable PGx implementation and research Provide guidance for appropriate feedback mechanism for PGx results Ensure laboratories comply with accreditation standard Govern ethical legal and social considerations for PGx research and implementation Authorize and standardize PGx testing specifications and reporting Provide guidelines for PGx medicine labelling Communicate with hospital leadership and payers Pharmacovigilance Serve as an advisory board	Governance Regulation Code of conduct
Healthcare champions (Kabbani et al., 2023)	Secure funds and infrastructure Lead the process and identify early adopters of change Ensure compliance with ethical, legal and social issues Monitor and evaluate the program’s impact Contribute to and lead new policies Communicate with regulatory bodies, payers, and pharmacy and therapeutics committee	Implementation Education
Pharmacy and therapeutics committee (Rigter et al., 2020; Kabbani et al., 2023)	Evaluate the available evidence in consultation with PGx consortia and networks Review the most relevant medicine with PGx considerations Consider the most actionable drug-gene pairs with related variant alleles for initial implementation Monitor and evaluate the PGx program for improvement Communicate with hospital leadership, laboratory, information technology and healthcare providers Collaborate on national guidelines on PGx	Implementation
Laboratory (Kabbani et al., 2023)	Perform genotyping/phenotyping/PGx testing analysis and reporting Collaborate to implement reactive point of care or pre-emptive testing Compare single vs. multiple gene testing Communicate with pharmacy and therapeutics committee, information technology and healthcare providers Provide expertise to interpret PGx results	Implementation Research
Information Technology (Rigter et al., 2020; Kabbani et al., 2023; Smith et al., 2025)	Report and integrate results into electronic health records for patients to benefit from continuity of care which should be accessible to authorized users and patients Design clinical decision support systems Communicate with pharmacy and therapeutics committee, laboratory, and healthcare providers Develop clinical decision support	Clinical decision support Research
Healthcare provider/Physician (Kabbani et al., 2023)	Order the test Understand and interpret the test results Make evidence-based therapeutic decisions Educate the community Communicate with patients, pharmacy and therapeutics committee, laboratory, and information technology	Implementation Education
Pharmacist (Haidar et al., 2022; Gammal et al., 2022; Kabbani et al., 2023)	Conduct practice-based research Lead implementation Educate healthcare professionals/community/patients Provide PGx counselling	Implementation Education Research
Genetic counsellor (Klein et al., 2017; Kabbani et al., 2023)	Provide patient counselling Collaborate with interprofessional team	Counselling
Patient (Kabbani et al., 2023)	Provide feedback on medication outcome Communicate with payers and healthcare providers Sharing of patient stories Patient advocates	End user perspective
Payer/Medical insurance companies (Rigter et al., 2020; Kabbani et al., 2023)	Conduct pharmaco-economic studies to consider re-imbursement for PGx testing Communicate with regulatory bodies, hospital leadership and patients Define prioritized conditions for PGx testing and reimbursement through health technology assessment	Implementation
Higher education institution (Rigter et al., 2020; Smith et al., 2025; Twesigomwe et al., 2025)	Conduct clinically relevant research Develop competency and curricula for healthcare professionals Educate healthcare professionals/ healthcare professional students as part of capacity building (global mindset, use of resources) Collaborate with industry/healthcare institutions/other universities Health technology assessment	Research Education
Professional bodies/societies (Rigter et al., 2020; Twesigomwe et al., 2025)	Advocate for the role of the profession in PGx Endorse competencies Develop context specific guidelines for PGx collaboration Knowledge and resource sharing Capacity building	Advocacy
		Endorsements
Policymaker (Alimohamed et al., 2025; Twesigomwe et al., 2025)	Develop implementation policies across Africa Provide evidence-based recommendations for testing/re-imbursement Standardize reporting Govern PGx research	Implementation Governance
Public health experts (Alimohamed et al., 2025; Borbon et al., 2025; Shaaban and Ji, 2023)	Recommend PGx for relevant public health conditions Conduct research Prioritize public health focus areas Health technology assessment	Research Implementation
Medical geneticist (Jongeneel et al., 2022; Twesigomwe et al., 2025)	Disease specific genetic testing	Implementation Research
Ethical and legal committees (Warnich et al., 2011; de Vries et al., 2017; Academy of Science of South Africa, 2018; Tata et al., 2020; Haskins, 2021; Twesigomwe et al., 2025)	Ethical legal and social implications of PGx implementation – return of patient results guidance Govern research	Oversight Governance
Healthcare institutions (Rigter et al., 2020; Twesigomwe et al., 2025)	Collaborate on national guidelines for PGx Provide PGx practice-based experience for healthcare professionals and students Health technology assessment	Implementation Research

### 2.3 Alignment of pharmacogenomics implementation with national health priorities

The integration of PGx into the South African healthcare system represents a transformative opportunity to enhance patient outcomes and support the government’s strategic goal of Universal Health Coverage (UHC). Aligning with the National Health Insurance (NHI) (Department of Health, 2024) framework and the Standard Treatment Guidelines (STGs), the clinical implementation of PGx could contribute to a stronger healthcare system by enabling more precise, individualized treatment strategies that could reduce adverse drug reactions, improve therapeutic efficacy, and optimize resource allocation. These objectives align with South Africa’s commitment to United Nations Sustainable Development Goal (SDG) 3B (United Nations, 2024) and Vision 2030, as outlined in the National Development Plan (South African Government, 2011). Integration with NHI Phase 1 (2025–2026) should include PGx pilots for high-cost ADR medicines (e.g., clopidogrel), directly supporting SDG 3B targets. Another recommended approach is to focus on high-risk populations e.g., pediatrics, HIV, TB patients (Nyame et al., 2024). The Department of Health’s Strategic Plan (2020/21–2024/25) (Department of Health, 2020) emphasizes the need to strengthen the healthcare system and improve the quality of care and priorities that PGx directly supports. Integrating PGx into clinical practice, regulatory frameworks, pharmacy services, genetic counselling, and professional training will not only advance precision medicine but also align with these national health priorities. In order to this, there is a need for health technology assessments to be performed to facilitate clinical implementation of robust and evidence-based PGx practices (National Human Genome Research Institute, 2024). During health technology assessment, cost benefit, cost utility and cost effectiveness may be considered (Ayati et al., 2021; Bardey et al., 2024). Many studies have explored cost effectiveness and cost utility of PGx. A recent systematic review reported that 71% (77/108) of the included studies indicated cost effectiveness (48/77) or cost saving (29/77) of PGx testing for medicines with Clinical Pharmacogenomics Implementation Consortium guidelines (Morris et al., 2022). The included studies were mostly from North America (47%), Europe (24%), Asia (26%) and Australia (3%). Patel and colleagues (Patel et al., 2025) reported on the cost effectiveness and/or cost saving of PGx testing per disease category in the US. Depression represented the greatest category, accounting for 43% of studies, followed by cardiovascular disease (22%), adverse drug reactions (14%) and pain management (14%). Due to the genetic diversity in Africa, PGx testing may have the potential for greater impact. Cost benefit analysis may particularly be beneficial for policy level decision making.

### 2.4 Framework for pharmacogenomics implementation in South Africa

To ensure the implementation and sustainability of PGx in South Africa, the authors call for the development of a pragmatic PGx and actionable framework for sustainable implementation. The proposed framework components (Table 2) were selected based on commonly reported barriers to PGx implementation globally, including lack of infrastructure, insufficient workforce capacity, regulatory gaps, and absence of sustainable financing models, challenges that are amplified in resource-constrained and genetically diverse settings such as South Africa. These components function interdependently: regulatory alignment enables reimbursement and ethical governance; infrastructure and informatics support laboratory testing and clinical decision support; workforce education enables interpretation and implementation; and stakeholder engagement ensures integration into routine clinical workflows. Sustainable implementation therefore depends on coordinated system-level integration rather than isolated interventions. The framework aims to be comprehensive and summarizes critical aspects, as well as identifies relevant role players. Existing evidence-based frameworks for PGx implementation can support context specific and local implementation of sustainable PGx practices (Aarons et al., 2011; Kreeftenberg et al., 2025). Local PGx leadership through experts and healthcare champions is a critical pillar for implementation and sustainable services of PGx. International stakeholders and partners in PGx can provide valuable input in the development and application of this critical, locally relevant framework while PGx research in genetically diverse populations such as South Africa can add tremendous value to global genetics data. Engagement with global experts can support implementation of localized best practices. Further stakeholder engagement at all levels is needed for sustainable PGx implementation, including these role players from the exploration phase is crucial for continuous support and to optimize resources. The proposed framework further outlines strategies for context-specific education, research, and infrastructure development essential for advancing local PGx implementation. Knowledge and information sharing (Radouani et al., 2020; Twesigomwe et al., 2025) is also vital to advance PGx in South Africa. Collaborative efforts among healthcare providers, researchers, policymakers, and patients are critical to ensure the responsible and equitable application of clinically relevant pharmacogenomics. The Academy of Science of South Africa (ASSAf) (Academy of Science of South Africa, 2018) emphasizes the need for governmental support in PGx implementation and policies for PGx implementation and research. An oversight committee should ensure responsible PGx testing, laboratory accreditation, and ensure that healthcare professionals have the required education in PGx. A national policy should provide guidance on providing feedback to individuals after PGx testing. Finally, a policy to ensure stewardship of research, data and clinical implementation is of utmost importance. Patient advocates and representatives from the community should be included to represent the patient’s voice. Table 2 provides the proposed framework considering various concepts that builds on the principles of Ubuntu. Ubuntu is an African philosophy and worldview rooted in the idea of shared humanity and interconnectedness.

**TABLE 2:** Proposed framework concept and stakeholder responsibility.

Framework concept	Stakeholder
PGx leadership	Special interest groups/working groups to champion PGx Healthcare professional champions to lead PGx implementation Professional bodies to provide frameworks for implementation Boards of experts providing guidance on implementation, research, education Healthcare authorities, policymakers Collaboration with global PGx experts Academic institutions
Stakeholders’ engagement for sustainability (Gurwitz et al., 2005; Shaaban and Ji, 2023)	Policymakers Public and private representatives Professional societies Regulatory bodies Hospital therapeutic committees Information technology PGx Healthcare champions Community representatives including traditional healers and patient representatives/advocates Higher education instutitions
Regulations (Frueh et al., 2005; Academy of Science of South Africa, 2018; Shaaban and Ji, 2023)	Laboratories/industry involved in PGx testing Medication manufacturing companies Healthcare institutions Healthcare professionals Higher education institutions Patient representatives
Context-specific research to strengthen implementation (Wells et al., 2008; Drozda et al., 2013; Dong et al., 2018; Radouani et al., 2020; da Rocha et al., 2021; Shaaban and Ji, 2023; Twesigomwe et al., 2025)	Affordable next-generation sequencing (NGS) to advance PGx research in South Africa NGS data must be publicly accessible to authorized users and in line with POPIA Research to identify local variants/genotypes to inform evidence-based medicine Diverse research teams
Education	Healthcare professionals – medical, pharmacy, nursing, genetic counsellors, laboratory personnel Healthcare professional students Public Patients
Interprofessional collaboration to advance PGx research, implementation and ensure sustainability of PGx (Warnich et al., 2011; Just et al., 2019; Shaaban and Ji, 2023)	Healthcare professionals Healthcare professional students Industry Higher education institutions Healthcare institutions Professional societies Patient representatives
Infrastructure support (Radouani et al., 2020; Twesigomwe et al., 2025)	Capacity building for effective and cost effective PGx testing in South Africa and broader Africa Bioinformatics and informatics

## 3 Call for action

Pharmacogenomics has the potential to improve patient health outcomes in South Africa and Africa. This call-to-action (Table 3) urges policymakers, healthcare professionals, healthcare and academic institutions, and industry stakeholders, including public and private healthcare laboratories and commercial partners, to collaborate in the adoption and implementation of PGx as a critical tool for achieving equitable, high-quality and safe healthcare for all South Africans. The action plan focuses on three critical pillars: research, implementation and education. Research should inform implementation but occur parallel to implementation to ensure evidence-informed practices. Research should focus on context specific next-generation sequencing to determine relevant genes and alleles for prioritization. Collaborative research among academic and healthcare institutions should be highly considered. Pharmacoeconomic studies should be conducted to support pre-emptive PGx testing where relevant. Evidence should inform the development of protocols for PGx testing for South Africa. A stepwise approach is recommended for PGx implementation. Local PGx experts are key informants in the development of a national PGx stewardship program. Initial research should focus on context specific and actionable gene-drug pairs. The success of PGx implementation is dependent on education focused on the multidisciplinary team, the patient and the public. Regulatory authorities play an important role in regulating PGx education. Higher education institutions should prioritize PGx education for healthcare professional students, while professional societies should offer continuing education opportunities for healthcare professionals in practice.

**TABLE 3:** Call for action at a glance.

Research	Implementation	Education
South Africa’s unique genetic diversity necessitates locally relevant research: • Prioritize studies on population-specific genetic variations affecting drug metabolism • Perform research in parallel to implementation to offer evidence-based practices • Integrate genomic initiatives to capture SA-specific alleles, prioritizing high-burden therapeutics	To ensure successful integration of PGx into clinical practice, we propose a tiered implementation plan: • Phase 1 (2025–2026): National PGx Stewardship Program development • Phase 2 (2027–2029): Launch pilot programs focusing on clinically relevant actionable gene-drug tertiary hospitals with existing molecular testing capacity. Validate cost-effectiveness using NHI ADR savings metrics to justify pilot drug selection (e.g., clopidogrel) • Phase 3 (2029–2030): Expand PGx testing of high impact medicines from phase 2, to primary care clinics using telehealth support systems and mobile testing units to reach rural areas • Phase 4 (2030 onwards): Scale nationwide adoption through partnerships with public-private entities under the NHI framework • This phased approach will allow for continuous evaluation of outcomes while addressing logistical challenges incrementally	Education is critical to empower healthcare professionals to implement PGx effectively: • Embed PGx education into healthcare professional accreditation requirements (HPCSA, SANC, SAPC), using case-based learning and interprofessional education including global comparisons • Develop continuing professional development programs for practicing healthcare professionals focused on interpreting PGx test results and applying them in clinical decision-making • Create contextualised awareness among SA population, including healthcare professionals on PGx to make informed decisions
Collaborative research efforts between academic institutions, government agencies, and industry will accelerate progress while ensuring sustainability	South Africa’s existing infrastructure provides a foundation for PGx implementation, but would require continuous coordination of the services: • Leverage the National Health Laboratory Service (NHLS) molecular testing networks established for HIV programs to support NGS for PGx research • Develop regional hub-and-spoke models where centralized labs conduct genomic analysis while smaller clinics collect samples • Incorporating cost-benefit analyses will strengthen advocacy efforts by demonstrating potential NHI savings from reduced ADR-related hospitalizations	Establish interprofessional training workshops that involve pharmacists, geneticists, nurses, genetic counsellors and doctors to foster collaboration
Ethical, Legal and Social Implications		
Given South Africa’s history of inequities in genetic research, ethical considerations must be central to its implementation: • Adopt local frameworks such as the H3Africa’s framework, which are guided by internationally accepted, local stakeholder informed insights. H3Africa’s community engagement framework, informed consent processes tailored for low-literacy populations, and benefit-sharing mechanisms (H3Africa Community Engagement Working Group for the Human Heredity and H3Africa Consortium September, 2017). The EPIS (Exploration, Preparation, Implementation, Sustainment) Framework provides a roadmap for context specific PGx implementation (Aarons et al., 2011; Moullin et al., 2019; Varughese et al., 2022) • Develop context specific policies informed by the Human Genetics and Genomics in South Africa: Ethical, Legal and Social Implications report (Academy of Science of South Africa, 2018) • Establish clear data ownership policies that protect indigenous genetic data from exploitation while promoting open-access research within ethical boundaries • These safeguards will ensure trust among communities while fostering equitable participation in PGx research initiatives		
To address rural-urban disparities in access: • Deploy mobile testing units equipped with point-of-care genotyping technologies to underserved areas • Engage genetically diverse communities through sustained stakeholder management and inclusive education and awareness campaigns • Establish telehealth consultation services where specialists can guide clinicians in remote areas on interpreting test results • Develop metrics to track adoption rates across socioeconomic groups to ensure equitable access • Apply Ubuntu principles through community-led consent protocols and data sovereignty agreements, ensuring indigenous data ownership via H3Africa or relevant governance models		

Due to South Africa’s political background, ethical legal and social implications should also be carefully considered. PGx testing should be available to all South Africans regardless if they are treated in the public or private sector. To address this sensitive topic, experts in ethical aspects should be included in the core stewardship team to provide the necessary guidance. In addition, patients should also be included in the design of research projects supporting PGx implementation and practices. Including the community in PGx research can be valuable to determine the PGx jargon that patients understand, how to provide feedback on PGx results and the disclosure of information (Claw, et al., 2024; Nabukenya, et al., 2024). Community engagement is an important factor to be considered in the stewardship program to address other social determinants of health. As pointed out by Shaaban and Ji (2023) PGx implementation strategies should include perspectives from underserved or disadvantaged communities to ensure inclusivity. To address social determinants of health such as socioeconomic status, language barriers, access to equitable PGx testing and mistrust in medicine, engagement with regulatory authorities, policymakers, pharmacy and therapeutics committee, as outlined in the framework, are proposed.

## 4 Conclusion

Pharmacogenomics offers South Africa an unprecedented opportunity to advance equitable, patient-centered healthcare by reducing adverse drug reactions, optimizing therapeutic outcomes, and supporting the goals of Universal Health Coverage. Yet, realizing this promise requires coordinated stewardship: locally relevant research, phased implementation, and education that empowers healthcare professionals and communities alike. By embedding ethical, legal, and social safeguards and embracing interprofessional collaboration, South Africa can lead the continent in responsible PGx integration. This call to action is not only about innovation in medicine, but about affirming the principles of Ubuntu, ensuring that the benefits of precision medicine are shared widely and justly, for the health of all.
